# Polarization of Hepatic Macrophages in Alveolar Echinococcosis and Its Role in Remodeling the Immune Microenvironment

**DOI:** 10.3390/tropicalmed11040096

**Published:** 2026-04-03

**Authors:** Hai Xu, Yanxiong Wang, Lin Mi, Li Ren, Zhixin Wang

**Affiliations:** Department of Hepatobiliary and Pancreatic Surgery, Qinghai University Affiliated Hospital, Xining 810000, China

**Keywords:** alveolar echinococcosis, macrophage polarization, immune microenvironment, immune evasion, immunotherapeutic strategies

## Abstract

Alveolar echinococcosis (AE), caused by *Echinococcus multilocularis* larvae, is a severe zoonotic disease mimicking tumors, primarily affecting the liver with high mortality if untreated. Host immunity plays a pivotal role, shifting from Th1/Th17-mediated clearance to Th2/Treg-driven tolerance, enabling parasite survival. Liver macrophages, including Kupffer cells, polarize towards M2 phenotype under parasite antigens (e.g., phytic acid, exosomes), promoting immunosuppression, fibrosis, and T cell exhaustion via IL-10/TGF-β. This reshapes the tumor-like immune microenvironment with M2 macrophages recruiting Tregs, suppressing NK/DC functions, and fostering angiogenesis/fibrosis. Current treatment remains centered on surgery and benzimidazole therapy, both of which have notable limitations. Experimental immunomodulatory strategies, drug repurposing approaches, and targeted delivery systems may offer future therapeutic opportunities, but these concepts remain largely preclinical, unproven in AE, and require careful evaluation for safety and efficacy.

## 1. Introduction

Alveolar echinococcosis (AE) is a severe zoonotic parasitic disease caused by the larvae of *Echinococcus multilocularis*, primarily affecting the liver and known as a “*parasitic tumor*” [1,2]. The lesions exhibit infiltrative growth within the liver, resembling malignant tumors, with the potential to invade adjacent tissues and metastasize distantly; without timely treatment, the mortality rate reaches up to 90% within 10–15 years [3,4,5]. Alveolar echinococcosis requires aggressive therapy. If feasible, curative treatment is a combination of radical surgical resection and albendazole chemotherapy; otherwise, lifelong albendazole is indicated [6]. By contrast, cystic echinococcosis (caused by *E. granulosus*) typically presents as well-encapsulated cysts that can often be managed by surgery or percutaneous aspiration and albendazole, following WHO stage-specific guidelines [7]. These differences underscore the poorer prognosis of AE and its requirement for prolonged treatment.

Due to the insidious nature of early symptoms, many patients are diagnosed at an advanced stage, rendering surgical resection unsuitable and necessitating long-term pharmacological management. The current first-line medications are benzimidazole anthelmintics (primarily albendazole), which exert only parasitostatic effects and are difficult to completely eradicate the parasite, requiring lifelong administration and prone to adverse effects such as hepatotoxicity [8,9]. Consequently, alveolar echinococcosis has been designated by the World Health Organization as one of the neglected tropical diseases requiring urgent control, underscoring the imperative for more effective therapeutic strategies [9,10,11].

The host immune response plays a decisive role in the disease progression of alveolar echinococcosis (AE). Studies indicate that the majority of individuals exposed to parasite eggs can achieve immune-mediated self-clearance or prevent parasite dissemination, with only less than 1% of infected individuals progressing to chronic disease due to immune dysregulation [12]. The larvae of *E. multilocularis* can persist long-term in the host, benefiting from induced immune modulation and evasion mechanisms, including macrophage polarization, T-cell exhaustion, and infiltration of immunosuppressive cells [13,14,15]. In recent years, understanding of the AE immune microenvironment has deepened, with particular attention to the functional shaping of host hepatic macrophages (including Kupffer cells and monocyte-derived macrophages). Macrophages, due to their plastic phenotypes, play a critical role in infection immunity: they can be activated into the M1 type that kills pathogens or polarized into the M2 type that promotes tissue repair and immunosuppression [16]. *E. multilocularis* may create an “immune-privileged” microenvironment conducive to its survival by regulating macrophage polarization [17]. This article focuses on the latest research progress regarding hepatic macrophage polarization and immune microenvironment remodeling mechanisms under AE infection, exploring their interactions and potential therapeutic strategies. By systematically reviewing cutting-edge achievements in this field, it aims to provide references for novel approaches in AE immunotherapy.

## 2. Immunological Basis of Alveolar Echinococcosis Infection

### 2.1. Overview of Host Immune Response

Upon entry into the host, *E. multilocularis* initially triggers an innate immune response, involving the recruitment and activation of macrophages, dendritic cells, neutrophils, and others. These innate immune cells, upon recognizing parasite antigens, produce pro-inflammatory cytokines such as IL-1β, IL-12, and TNF-α, initiating an adaptive immune response dominated by Th1/Th17, which restricts early parasite growth [18,19]. In immunocompetent hosts, the initial Th1/Th17 response typically effectively controls or clears the parasite: for instance, IL-12 and IFN-γ activate the cytotoxic functions of macrophages and NK cells, while IL-17 mobilizes neutrophils to participate in anti-parasitic activities [12,20]. Studies estimate that in endemic areas, approximately 99% of infected individuals’ immune systems can terminate the infection at early lesions or form degenerative cysts without progression [12].

### 2.2. Immune Tolerance and Disease Progression

**Figure 1:**
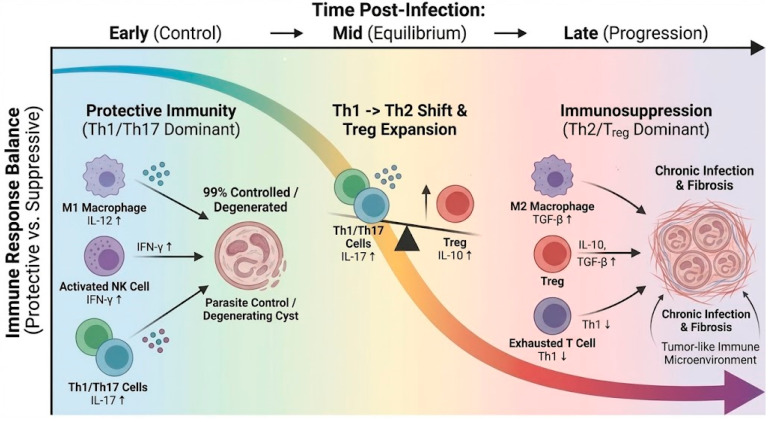
Schematic Timeline of Immune Response in Alveolar Echinococcosis Infection. Figure Legend: this figure illustrates the temporal changes in the host immune response during alveolar echinococcosis (AE) infection. In the early stage of infection, the host primarily employs Th1/Th17-mediated immune responses for antiparasitic defense, characterized by elevated levels of pro-inflammatory cytokines such as IL-12, IFN-γ, and IL-17, with the immune response dominated by M1-type macrophages. During this phase, the majority of infected individuals successfully control the parasite through the immune system, preventing disease progression. As the infection persists, the immune response gradually shifts toward Th2/Treg reactions, with an increase in immunosuppressive factors such as IL-10 and TGF-β, and the immune response dominated by M2-type macrophages, leading to the development of immune tolerance. Ultimately, AE progresses to the chronic stage, where the parasite evades immune clearance through mechanisms such as macrophage polarization and T-cell exhaustion, forming a tumor-like immune microenvironment that consequently results in chronic infection, fibrosis, and immune escape (Top horizontal arrows indicate temporal progression. Black arrows indicate the direction of immune effects or stage transitions. Upward arrows indicate increased cytokine expression/activity).

In a minority of susceptible hosts, the immune response shifts from Th1 to Th2, accompanied by the expansion of regulatory T cells (Treg), leading to parasite evasion of immune clearance and entry into a chronic infection phase [15,21]. Peripheral blood of AE patients often exhibits elevated Th2 and Treg-associated cytokines, such as IL-10 and TGF-β, while pro-inflammatory Th1 cytokines are suppressed, indicating a gradual entry into a state of immune tolerance [13,22]. Treg actively downregulates protective inflammatory responses through the secretion of IL-10 and TGF-β, considered one of the primary driving factors in establishing chronic echinococcal infection [23]. Concurrently, the host produces high-titer specific IgG and IgE antibodies, but the presence of vesicular structures renders humoral immunity ineffective in directly clearing the parasite. Overall, the immune landscape of AE manifests as an early phase dominated by Th1/Th17-mediated anti-parasitic immunity, transitioning to Th2/Treg-mediated immunosuppression in the later phase, with the tilt in this balance determining the infection outcome [12]. This unique biphasic immune response serves as the foundation for understanding the pathogenesis of AE. As summarized in Figure 1, AE is characterized by a temporal shift from an early Th1/Th17-dominant antiparasitic response toward a later Th2/Treg-skewed immunotolerant state. This schematic integrates the dynamic transition of cytokine profiles, macrophage polarization, and immune escape, thereby providing a concise framework for understanding stage-dependent immune remodeling during AE progression.

### 2.3. “Tumor-like” Immune Microenvironment

In advanced AE lesions, the parasite and host immune system achieve a dynamic equilibrium: the immune system is insufficient to eradicate the parasite but can restrict its growth rate, while the parasite induces the host to form a local immunosuppressive microenvironment through various mechanisms to achieve “long-term coexistence with the parasite” [23]. This microenvironment shares numerous similarities with the tumor immune microenvironment, which is why AE is often likened to a “parasitic cancer” [2,24]. For example, substantial macrophage and lymphocyte infiltration can be observed around AE lesions, but a significant portion consists of M2-type macrophages, Treg, and T cells with exhausted phenotypes, which secrete factors such as IL-10 and TGF-β, suppressing effective anti-parasitic immunity [22]. Simultaneously, the parasite continuously stimulates granulation tissue to undergo fibrosis and neovascularization, providing itself with shelter and nutritional supply [17]. It can be said that the immunological basis of AE lesions largely manifests as a “cold inflammation” environment shaped by the long-term interplay between host and parasite: encompassing both the progression of chronic inflammation and tissue damage, as well as the maintenance of immune evasion and tolerance mechanisms.

To sum up, in early AE infection, a Th1/Th17-driven response can contain the parasite, whereas progression to chronic disease involves a shift to Th2/Treg dominance that permits parasite persistence. The balance between protective inflammation and immune tolerance is thus decisive for AE outcomes.

## 3. Mechanisms of Hepatic Macrophage Polarization

### 3.1. Concept of M1/M2 Polarization in Macrophages

Hepatic macrophages include resident Kupffer cells and monocyte-derived infiltrating macrophages, playing a central role in liver immune homeostasis and diseases. Depending on different stimuli from microenvironmental signals, macrophages can undergo functional phenotypic polarization. One end is the classically activated M1-type macrophages, triggered by interferon-γ (IFN-γ), lipopolysaccharide (LPS), and others, activated via signaling pathways such as STAT1 and NF-κB, producing high levels of mediators like IL-12, IL-1β, TNF-α, and inducible nitric oxide synthase (iNOS), possessing strong pathogen-killing and pro-inflammatory effects [25]. The other end is the alternatively activated M2-type macrophages, induced by Th2-type cytokines such as IL-4 and IL-13, mediated through pathways like STAT6 and PPARγ, expressing arginase-1 (Arg-1), mannose receptor CD206, anti-inflammatory factors IL-10 and TGF-β, with primary functions of suppressing inflammation, promoting tissue repair, and fibrosis [26,27]. It is important to emphasize that M1/M2 represent two extreme functional spectra, and in actual physiological and pathological conditions, macrophages often exhibit diverse phenotypes on a continuous spectrum, with certain overlaps and reversible transitions [28]. However, for research convenience, the M1/M2 framework is still widely used to describe the two typical functional states of macrophages in inflammation and immunity.

### 3.2. Molecular Mechanisms of Polarization Regulation

**Figure 2:**
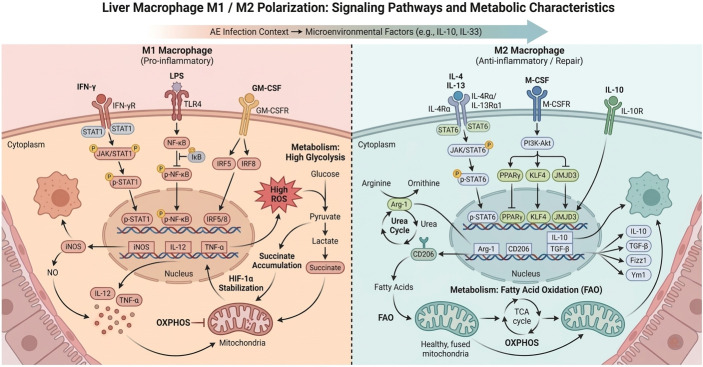
Signaling Pathways and Metabolic Features of M1/M2 Polarization in Hepatic Macrophages. Figure Legend: this figure compares the polarization mechanisms and metabolic features of M1-type and M2-type hepatic macrophages. M1-type macrophages are primarily activated by factors such as IFN-γ, LPS, and GM-CSF, enhancing pro-inflammatory responses through signaling pathways like STAT1 and NF-κB, producing pro-inflammatory factors including iNOS, IL-12, and TNF-α, and relying on the glycolytic pathway to provide energy for their pathogen-killing functions. M2-type macrophages, under stimulation by Th2-type cytokines such as IL-4 and IL-13, polarize through signaling pathways including STAT6, PI3K-Akt, and PPARγ, exhibiting high expression of immunosuppressive factors like Arg-1, CD206, IL-10, and TGF-β, with primary functions of promoting tissue repair and fibrosis. Furthermore, M1-type macrophages favor glycolytic metabolism, whereas M2-type macrophages support their immunosuppressive and reparative functions through fatty acid oxidation and OXPHOS metabolic pathways (Standard arrows indicate activation or signaling flow, T-shaped blunt lines indicate inhibition, vertical arrows indicate translocation, and curved arrows indicate metabolite flow).

Macrophage polarization is regulated by various signaling molecules. Key factors promoting M1 polarization include IFN-γ, interleukin-12 (IL-12), and granulocyte-macrophage colony-stimulating factor (GM-CSF), which induce M1-related gene expression, such as iNOS and IL-12p40 subunit genes, by activating transcription factors like STAT1, IRF5, and IRF8 within macrophages [16,29]. In contrast, factors promoting M2 polarization include IL-4, IL-13, and M-CSF, with their primary signaling pathways being STAT6 and PI3K-Akt, upregulating transcriptional regulators such as Kruppel-like factor 4 (KLF4) and JMJD3, thereby driving the expression of M2 marker genes like Arg-1, Fizz1, and Ym1 [30,31]. Additionally, IL-10, glucocorticoids, vitamin D3, and others in the microenvironment can also promote macrophage shifts toward M2 functions. Metabolic states are also closely related to polarization: M1 prefers glycolytic pathways for energy supply accompanied by decreased mitochondrial oxidative function, while M2 relies on mitochondrial oxidative phosphorylation and fatty acid oxidation to meet functional demands [32,33]. This immunometabolic reprogramming further consolidates the polarization state of macrophages. For example, high levels of the glucose metabolism intermediate succinate accumulate in M1 macrophages, amplifying inflammatory responses by stabilizing HIF-1α; whereas in M2 macrophages, enhanced fatty acid oxidation and ornithine cycle contribute to tissue repair and collagen production. Macrophage polarization is also subject to epigenetic regulation, such as histone modifications and non-coding RNA regulations, conferring a “memory” effect to the polarization state over a certain period. These complex mechanisms ensure that hepatic macrophages can dynamically adjust their phenotypic functions according to the constantly changing environmental signals during AE infection. Figure 2 provides an integrated comparison of the signaling pathways, effector molecules, and metabolic features associated with M1- and M2-like hepatic macrophage polarization. By combining immune signaling and immunometabolic reprogramming in a single schematic, this figure highlights the mechanistic basis by which macrophage functional states may differentially influence inflammation, tissue repair, and fibrosis in AE.

### 3.3. Role of Polarization in Liver Pathology

In liver diseases, macrophage polarization has a significant impact on the processes of inflammation and fibrosis. On one hand, M1 macrophages can directly inhibit pathogens and kill infected cells by secreting mediators such as TNF-α and IL-1β, but if the response is overly strong, it may also lead to tissue damage; On the other hand, TGF-α and TGF-β released by M2 macrophages can stimulate the activation of hepatic stellate cells (HSC) and promote collagen deposition, thereby driving liver fibrosis [34]. In models such as non-alcoholic steatohepatitis and mouse liver resection regeneration, observations show that temporal changes in the M1/M2 macrophage ratio are closely related to disease progression and recovery. For instance, M1 dominates in the early injury phase to clear necrotic tissue, followed by an increase in M2 to promote tissue repair [35,36,37]. For alveolar echinococcosis, a chronic liver infection, macrophage polarization imbalance may result in an inability to effectively eliminate the parasite while promoting lesion fibrosis and immune evasion [38,39,40].

Hepatic macrophage polarization in AE should be viewed as a dynamic and reversible spectrum rather than a rigid M1/M2 dichotomy. Parasite-derived signals and host microenvironmental cues jointly bias macrophages toward an immunosuppressive and pro-fibrotic phenotype, which may contribute to parasite persistence and lesion progression. A clearer understanding of this plasticity is essential for interpreting pathogenic mechanisms and identifying therapeutic targets.

## 4. Interaction Between Alveolar Echinococcus and Macrophage Polarization

### 4.1. Parasite Antigen-Induced Macrophage Polarization

Recent research evidence indicates that alveolar echinococcosis infection can significantly drive host macrophages toward an M2 phenotype shift. For example, Chong et al. [16]; stimulated mouse macrophage cell lines RAW264.7 and Ana-1 in vitro with soluble antigens from *E. multilocularis*, observing significant upregulation of M2 markers Arg-1 and IL-10 within just 8 h, while M1 markers IL-12 and iNOS levels were downregulated. This suggests that parasite antigens directly promote macrophage polarization into the immunosuppressive and repair-promoting M2 subtype. Simultaneously, the study found that co-cultured macrophages upregulated proteins related to the RhoA-ROCK and MAPK signaling pathways, and the application of RhoA/MAPK pathway inhibitors could partially reverse the M2 polarization tendency of macrophages. Notably, when these parasite antigen-stimulated M2-like macrophages were co-cultured with hepatic stellate cells, the latter exhibited typical myofibroblast transdifferentiation (increased α-SMA expression), suggesting that parasite-induced macrophages can promote liver fibrosis [16]. Another study co-cultured mouse bone marrow macrophages with *E. multilocularis* cyst fluid or protoscoleces (protoscolex), where the proportion of M2-polarized macrophages was significantly higher than M1, manifested as upregulation of M2-related genes such as Arg-1 and CCL22, while M1 markers like iNOS showed no significant changes. Simultaneously, the expression of key enzymes in the glycolytic pathway (such as HK and PFK) in these co-cultured macrophages increased, with *E. multilocularis* inducing macrophage metabolism toward a high glycolytic state via the PI3K/Akt/mTOR signaling pathway, thereby leading to M2-type polarization of macrophages. Glycolytic metabolic reprogramming may be a crucial link in parasite-induced changes in macrophage function: sufficient glycolytic energy supply might aim to meet the needs of M2 macrophages for secreting matrix remodeling factors (such as collagen), or reflect the dynamic balance of macrophages between inflammatory and immunosuppressive functions. In summary, the antigen molecules released by *E. multilocularis* not only directly promote phenotypic shifts in macrophages but also profoundly influence their metabolic and signaling networks, thereby shaping cellular functional states conducive to parasite survival [30,33].

### 4.2. Parasite-Derived Molecule-Mediated Immune Regulation

**Figure 3:**
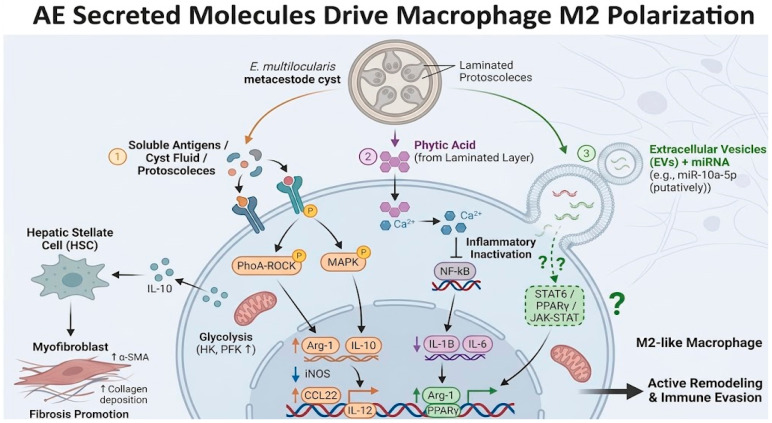
Schematic Diagram of the Mechanism by Which Alveolar Echinococcus Secreted Molecules Drive M2 Polarization of Macrophages. Figure Legend: this figure illustrates the mechanism by which alveolar echinococcosis induces M2 polarization of host macrophages through secreted antigenic molecules and related factors. The parasite directly stimulates host macrophages by releasing soluble antigens, cyst fluid, or protoscoleces, activating RhoA-ROCK and MAPK signaling pathways, promoting the expression of M2 markers such as Arg-1 and IL-10, while inhibiting the upregulation of M1 markers such as iNOS and IL-12. Furthermore, the phytic acid secreted by the parasite chelates Ca^2+^ within macrophages, inhibiting the NF-κB signaling pathway, thereby attenuating pro-inflammatory responses and further promoting M2 polarization, Extracellular vehicles (EVs) containing miRNAs (e.g., miR-10a-5p, putatively) may target the STAT6/PPARγ/JAK-STAT pathway; this mechanism remains hypothetical and is depicted with dashed lines and question marks. These coordinated actions collectively drive macrophages toward an M2-like phenotype, resulting in active tissue remodeling, fibrosis promotion (via hepatic stellate cell activation and collagen deposition), and parasite immune evasion (Solid arrows indicate experimentally supported signaling or regulatory effects, T-shaped blunt lines indicate inhibition, dashed lines/dashed outlines indicate putative or incompletely validated mechanisms, and question marks indicate uncertain molecular targets or pathways).

*E. multilocularis* possesses a series of unique molecules that can actively intervene in the activation pathways of host macrophages. One typical example is phytic acid, which has been confirmed to be abundantly present in the laminated layer structure of the echinococcal cyst wall [41]. Salzmann et al. [41]; reported that phytic acid is an important “small molecule weapon” in parasite-mediated macrophage deactivation: in infected mouse livers, although macrophages accumulate extensively around echinococcal vesicles, they hardly express pro-inflammatory factors like IL-6, presenting a suppressed inflammatory phenotype. In vitro experiments further confirmed that phytic acid can chelate intracellular Ca^2+^ in macrophages, thereby reducing the cells’ ability to produce IL-1β and IL-6 under LPS+IFN-γ stimulation, with effects similar to directly adding parasite vesicle extracts. This indicates that echinococci can achieve “chemical” suppression of macrophage inflammatory responses through secreting non-protein factors like phytic acid, representing an immune evasion strategy independent of protein virulence factors [41]. Additionally, evidence from related parasites further suggests that secreted proteins and EV-associated miRNAs may modulate macrophage function. For example, antigen B in the cyst fluid of *E. granulosus* (*cystic echinococcosis*) can blunt macrophage M1 activation via Fcγ receptors [42]. Similarly, extracellular vesicles secreted by *Trichinella spiralis* containing microRNAs such as let-7-5p can promote macrophage conversion to the M2 phenotype [43]. While direct evidence for *E. multilocularis* EV-derived miRNAs in AE remains limited (research on EVs is in its infancy), analogous mechanisms from related parasites suggest that candidate miRNAs such as miR-10a-5p may target STAT6/PPARγ in host macrophages [44]. This remains a working hypothesis, supported by emerging in vitro data, but requiring functional validation in AE-specific models. In summary, *E. multilocularis* has evolved various secretory molecular weapons, from ion chelators to proteins/non-coding RNAs, continuously interfering with the normal immune functions of host macrophages, biasing them toward anti-inflammatory/repair states favorable for parasite persistence. Figure 3 summarizes how parasite-derived factors may collectively skew macrophages toward an M2-like phenotype through coordinated effects on signaling pathways and inflammatory responses. By integrating soluble antigens, phytic acid, and exosome-associated regulatory molecules into one model, the figure highlights the multi-layered mechanisms through which *E. multilocularis* may promote immune evasion and tissue remodeling.

### 4.3. Adaptive Changes in Host Macrophage Phenotypes

In addition to direct parasite effects, host factors during chronic infection also drive changes in macrophage phenotypes. For example, within AE lesions, due to persistent tissue damage by the parasite causing cell necrosis and stress, the host releases damage-associated alarmins, such as IL-33. It is a nuclear-cytoplasmic factor released upon damage to epithelial and endothelial cells, possessing the ability to drive Th2 immunity. Autier et al. [12]; revealed in AE mouse models that elevated IL-33 correlates with M2-like macrophage polarization and larger lesions. Notably, this evidence comes primarily from murine experiments; the role of IL-33 in human AE lesions remains to be confirmed, so extrapolations should be made with caution. Immunohistochemistry showed that IL-33 is primarily expressed by neovascular endothelial cells near parasite lesions and co-localizes with accumulated FOXP3+ Treg in these areas. IL-33 acts through its receptor ST2 on innate lymphoid cells, macrophages, and Treg in the stroma, inducing the secretion of more Th2 and suppressive cytokines, overall tilting macrophages and helper T cells toward immune tolerance [12]. Therefore, certain self-factors secreted by the host during chronic infection also play a role in reinforcing macrophage M2 polarization and microenvironmental immune suppression. In summary, in the context of *E. multilocularis* infection, parasite factors and host factors jointly shape the polarization state of macrophages: the former provides signals to actively deflect macrophage functions, while the latter’s inflammation–anti-inflammation feedback mechanisms further selectively amplify the number and activity of M2-phenotype macrophages. This host–pathogen synergy-induced adaptive change in macrophages forms the basis for the AE immune microenvironment. In summary, *E. multilocularis* can directly reprogram hepatic macrophages through parasite-derived molecules such as soluble antigens, cyst fluid/protoscolex components, exosomal miRNAs, and phytic acid, or indirectly promote the shift in macrophages toward the immunosuppressive and pro-repair M2 phenotype by inducing host factors (such as IL-33). For ease of comparative summarization of different inducing factors, key signaling pathways, and phenotypic effects, the molecular mechanisms by which *E. multilocularis* induces M2 polarization are compiled in Table 1.

## 5. Mechanisms of Immune Microenvironment Remodeling

**Figure 4:**
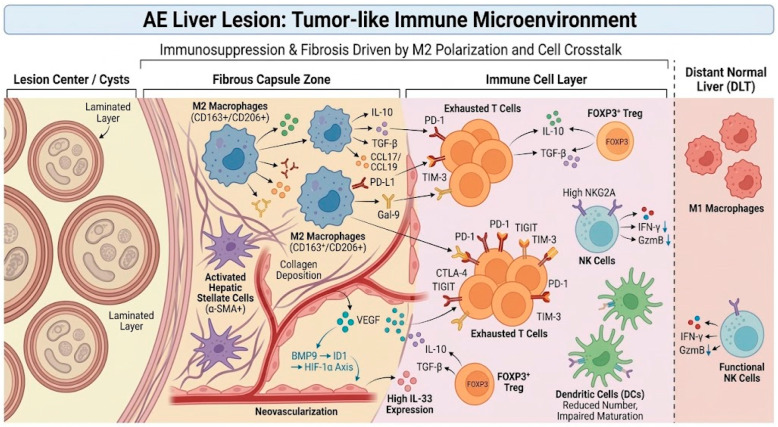
Schematic Diagram of the “Tumor-like” Immune Microenvironment Composition in Hepatic Lesions of Alveolar Echinococcosis. Figure Legend: this figure illustrates the “tumor-like” immune microenvironment in hepatic lesions of alveolar echinococcosis, collectively formed by parasite cysts, fibrous pericyst, and surrounding immune cells/matrix components. The lesion center consists of parasite cysts and their laminated layer structures, which can adsorb host immunoglobulins and complement regulatory molecules, forming a dual physical and molecular barrier against humoral immunity. Around the cysts, a thick fibrous pericyst forms, with accumulation of numerous M2-type macrophages (CD163^+^, CD206^+^) and activated hepatic stellate cells (α-SMA^+^); the former secretes factors such as IL-10, TGF-β, CCL17, CCL19, and PD-L1, while the latter produce collagen deposition, promoting liver fibrosis and lesion encapsulation. Neovascularization is abundantly generated in the pericyst area driven by factors such as VEGF, with endothelial cells highly expressing IL-33, providing signals for Th2 deviation and Treg enrichment. In the adjacent hepatic tissue of the lesion, a large number of exhausted phenotype T cells (with high expression of PD-1, CTLA-4, TIGIT, LAG-3, Tim-3), FOXP3^+^ Tregs, and NK cells with high NKG2A expression can be observed, while dendritic cell numbers are reduced and maturation is impaired. Overall, this microenvironment both restricts parasite dissemination and significantly suppresses effective antiparasitic immunity, providing an “immune sanctuary” for the long-term survival of the parasite (Arrows indicate the direction of cellular crosstalk, cytokine/chemokine signaling, recruitment, or functional influence, whereas dashed boundary lines indicate spatial compartmentalization within the lesion microenvironment).

The “immune microenvironment” formed by *E. multilocularis* in the host is a complex network composed of various immune cells, cytokines, and matrix components, with prominent features of immune suppression and fibrosis. Changes in macrophage polarization occupy a central position therein, and through interactions with other immune cells, collectively remodel the local immune ecology. The following elucidates the remodeling mechanisms of the AE lesion immune microenvironment from cellular and molecular levels. As illustrated in Figure 4, the AE lesion microenvironment comprises a highly organized network of parasite structures, fibrotic stroma, neovasculature, and immunosuppressive immune cells. This integrative schematic is intended to visualize how macrophages, Treg cells, exhausted T cells, NK cells, dendritic cells, and stromal elements are spatially and functionally linked within the tumor-like immune niche of AE.

### 5.1. Role of Macrophages in the Microenvironment

It is important to note that macrophages in AE lesions are highly heterogeneous and plastic; rather than existing as purely ‘M1’ or ‘M2’, human tissue analyses suggest they display a continuum of activation states with overlapping markers and functions. Likewise, recent single-cell analyses further support immune heterogeneity in helminth-infected liver tissue. In murine hepatic AE, scRNA-seq identified macrophages among 11 major cell populations, although macrophage intermediate states were not fully resolved. Analogous scRNA-seq studies in schistosomal liver fibrosis have profiled distinct macrophage subsets, suggesting that helminth-associated hepatic macrophages exist along a dynamic continuum rather than as strictly discrete M1 or M2 populations [49]. But among the macrophages that accumulate in large numbers around AE lesions, the M2 phenotype predominates, and the mediators secreted by these cells have extensive effects on the microenvironment [40]. First, M2 macrophages produce high levels of immunosuppressive factors such as IL-10 and TGF-β, which can directly inhibit the antigen presentation capability of macrophages themselves and adjacent dendritic cells, reducing the latter’s function in triggering T cell responses, thereby weakening the local adaptive immune response [12,17]. This has been confirmed in clinical samples and mouse models: analysis of liver tissues from patients and mice revealed that in liver tissues close to parasitic lesions (CLT), although there is abundant infiltration of antigen-presenting cells (including macrophages/DCs), their activation markers and pro-inflammatory cytokine levels are lower than in distant normal liver tissues (DLT), suggesting that innate immune cells such as macrophages are functionally suppressed at the lesion site [22,50]. Second, chemokines secreted by M2 macrophages (such as CCL17 and CCL19) recruit more Treg and Th2 cells into the lesion, amplifying the immunosuppressive effects. Studies have examined the chemokine profiles in the serum and lesions of AE patients, finding that CCL17/CCL19 are significantly elevated and correlated with the degree of macrophage infiltration [51]. This indicates that macrophages not only act as immunosuppressors themselves but also actively shape the composition of surrounding lymphocytes through the secretion of chemotactic signals. Third, macrophages are one of the primary drivers of AE-related liver fibrosis [52]. M2 macrophages continuously release pro-fibrotic mediators such as TGF-β and PDGF, stimulating hepatic stellate cells to produce large amounts of collagen and extracellular matrix, thereby forming a thick fibrous capsule around the parasitic lesion. Fibrosis serves as a host measure to restrict parasite dissemination, but excessive fibrosis can also compress liver tissue and lead to liver function impairment [16]. In liver biopsies from AE patients, numerous α-SMA-positive activated stellate cells are observed, and these areas are often rich in CD163+ and CD206+ macrophages (M2 phenotype), with the two being spatially closely associated [40,53]. In summary, through dual pathways of immunosuppression and promotion of fibrosis, macrophages become the central “engineers” in reshaping the AE microenvironment. However, it remains unclear to what extent the predominance of M2 macrophages in AE lesions is a direct driver of the pronounced fibrosis and parasite persistence, or rather a consequence of the chronic infection milieu; current evidence is largely correlative, and this causality is an area of active investigation.

### 5.2. T Cell Exhaustion and Regulation

The adaptive immunity within AE lesions has also undergone significant remodeling, characterized by impaired T cell function. In the immunosuppressive environment of chronic infection, persistent antigen presence leads to effector T cells exhibiting an exhausted phenotype, with high expression of multiple inhibitory receptors such as programmed death receptor-1 (PD-1), cytotoxic T lymphocyte-associated antigen 4 (CTLA-4), TIGIT, Tim-3, etc. [14,54,55]. Sun et al. [22]; detected in AE patient liver tissues that, compared to distant normal liver tissues, CD4+ lymphocytes accumulate in large numbers in lesion-adjacent areas (CLT), with a significantly increased proportion of cells co-expressing PD-1 or CTLA-4. Flow cytometry further confirmed that these PD-1 or CTLA-4 expressing CD4+ T cells are primarily CD25 + Foxp3+ Treg and some exhausted effector T cells [22]. High expression of PD-1/CTLA-4 indicates that T cells are in a functionally suppressed or tolerant state, with significantly reduced proliferation and cytotoxic capabilities. This has been functionally validated in animal models: blocking the PD-1/PD-L1 pathway can partially restore the proliferation and IFN-γ secretion capabilities of exhausted T cells, and reduce parasite burden in mice [56]; similarly, CTLA-4 antibody blockade can enhance T cell activity and combat parasite infection [57]. Additionally, Treg cells are enriched in AE, where they not only exhaust effector T cell responses but also actively secrete IL-10 and TGF-β to enhance local immune tolerance [23]. The proportion of Treg in patients’ peripheral blood and lesions is elevated and positively correlated with disease severity. Therefore, in the AE microenvironment, the T cell pool is reshaped due to persistent antigen stimulation and inhibitory signals: useful anti-parasitic effector T cells become functionally exhausted and unable to clear the parasite; conversely, inhibitory Treg accumulate, further consolidating the immunosuppressive state of the microenvironment [58,59]. It is worth noting that macrophages and T cells interact mutually in this process: ligands such as PD-L1 and Gal-9 highly expressed by M2 macrophages bind to PD-1 and Tim-3 receptors on T cells, inducing T cell exhaustion and apoptosis [22,60]; conversely, IL-10 secreted by Treg stimulates macrophages to maintain the M2 phenotype, forming a positive feedback loop [40]. This synergy of the “macrophage-T cell axis” turns the AE lesion into a highly controlled immunosuppressive zone [17].

### 5.3. Changes in Innate Immune Cells

**Figure 5:**
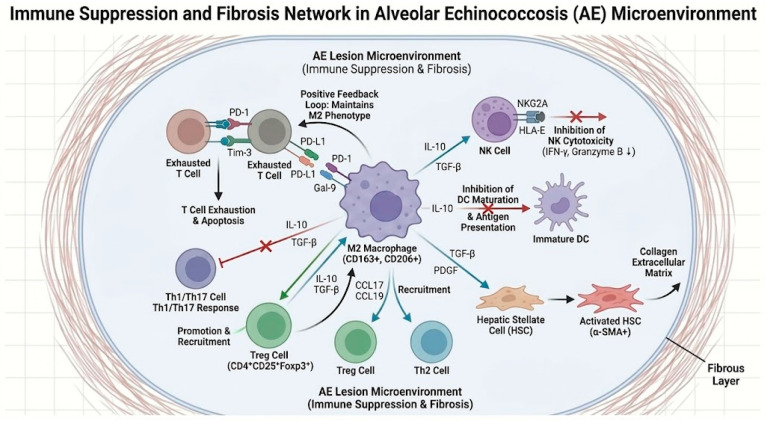
The “Macrophage–T Cell–NK Cell” Immunosuppressive Positive Feedback Network in AE. Figure Legend: this figure summarizes the “macrophage–T cell–NK cell” immunosuppressive positive feedback loop driven by hepatic macrophage polarization during alveolar echinococcosis infection. M2-type macrophages, through high expression of ligands such as PD-L1 and Gal-9, bind to receptors like PD-1 and Tim-3 on the surface of T cells, inducing functional exhaustion of effector T cells, while simultaneously secreting large amounts of IL-10 and TGF-β to suppress Th1/Th17 responses and promote Treg expansion. In turn, Tregs release factors such as IL-10 and TGF-β, further stabilizing the M2 phenotype of macrophages and forming immunosuppressive positive feedback between “macrophages–Tregs.” Furthermore, inhibitory cytokines secreted by M2-type macrophages can reduce the cytotoxic activity of NK cells and, by promoting activation of the NKG2A–HLA-E axis, drive NK cells into a hyporesponsive state; simultaneously, they inhibit dendritic cell maturation and antigen presentation capabilities, attenuating initial T cell activation. The aforementioned multiple feedback loops collectively maintain immune tolerance and a “cold inflammation” state in AE lesions, creating conditions for persistent parasite infection and progression of liver fibrosis, while also indicating that molecules such as PD-1/PD-L1, Tim-3, and NKG2A are potential targets for immune intervention (Standard arrows indicate activation, promotion, recruitment, or positive feedback interactions, whereas T-shaped blunt lines indicate inhibition or suppression).

In addition to macrophages, the functions of other innate immune cells in the AE microenvironment are also suppressed, further weakening anti-parasitic immunity. NK cells, as important innate immune effector cells, should exert cytotoxic effects in the early stages of parasitic infection. However, in AE patient livers, NK cells in perilesional regions also exhibit exhaustive changes [23]. Studies have found that NK cells in the CLT region exhibit high expression of the inhibitory receptor NKG2A on their surface, which is negatively correlated with significantly reduced secretion of IFN-γ and granzyme B; in contrast, NK cells in distant regions have low expression of the NKG2A inhibitory receptor and normal function. Upon binding to HLA-E, NKG2A transmits inhibitory signals, causing NK cells to “brake.” The proportion of NKG2A+ NK cells in AE patient lesions is increased, with reduced IFN-γ production, suggesting that the parasite may induce host high expression of HLA-E or similar ligands to keep NK cells silent [61]. The role of neutrophils in AE is relatively minor, but studies have reported a decline in their phagocytic and oxidative burst capabilities during the chronic infection phase [45,62]. Regarding dendritic cells, AE patients exhibit reduced numbers of monocyte-derived DCs in peripheral blood, with impaired maturation, possibly related to TLR signal tolerance induced by parasitic antigens [50]. These changes are inextricably linked to inhibitory factors released by M2 macrophages. For example, IL-10 can inhibit DC maturation and reduce NK cell activity; TGF-β can also suppress neutrophil activity [22]. It is evident that in the AE microenvironment, multiple components of innate immunity are reshaped into low-reactivity, tolerant states, collectively paving the way for the parasite’s persistent survival. Figure 5 integrates the reciprocal interactions among M2-like macrophages, exhausted T cells, Treg cells, NK cells, and dendritic cells in AE. By summarizing this macrophage–T cell–NK cell feedback network, the figure emphasizes how multiple suppressive pathways may cooperate to sustain immune tolerance, impair antiparasitic immunity, and facilitate chronic lesion persistence.

### 5.4. Remodeling of Stroma and Vasculature

Changes in the immune microenvironment are also reflected at the tissue structural level. AE lesions often form distinct fibrous encapsulations, accompanied internally by neovascular networks and lymphatic vessel proliferation [46]. Fibrosis results from the combined effects of immune responses and direct parasite actions: during inflammation, macrophages and Th2 cells promote stellate cells to produce collagen, while parasitic mechanical stimulation and secretions also induce fibroblast proliferation [53]. Neovascularization is driven by factors such as VEGF, providing blood supply to the parasite [48]; Alarmins such as IL-33 are overexpressed in the endothelial cells of these new vessels [12]. Furthermore, the structure of the alveolar echinococcus cyst wall itself (such as the laminated layer) participates in microenvironment remodeling: its strong negative charge and abundant glycosaminoglycans enable it to adsorb host complement regulatory proteins and immunoglobulins, thereby evading immune attacks [63]. This “molecular filtration” effect adds an immunosuppressive barrier to the microenvironment. Finally, due to the long-term presence of the lesion, a phenomenon known as “peripheral immune organs” can form locally, such as tertiary lymphoid follicle-like structures near the fibrous capsule, containing B cells and TFH cells [64]. Although anti-echinococcus antibodies can be detected in AE patients, these antibodies are mostly non-neutralizing, and the parasite has mechanisms (such as surface adsorption of host immunoglobulin fragments) to evade antibody effects [47]. These histological changes, together with alterations in immune cell functions, constitute the unique immune microenvironment of AE.

Overall, the AE lesion microenvironment is shaped by coordinated interactions among M2-skewed macrophages, exhausted T cells, Treg cells, NK cells, dendritic cells, and stromal components. These interactions collectively promote immune tolerance, fibrosis, and vascular remodeling, thereby restricting effective antiparasitic immunity while supporting long-term parasite survival. This integrated cellular network may represent a central mechanistic basis for the tumor-like behavior of AE lesions. The main components of the aforementioned AE lesion immune microenvironment and their roles are now summarized in Table 2.

## 6. Treatment Strategies and Prospects

### 6.1. Limitations of Existing Treatment Methods (Current Practice)

Traditionally, the primary treatment for AE is surgical resection of the lesion, combined with long-term postoperative albendazole medication to control residual parasites [65]. The prerequisite for surgery to achieve a radical cure is that the lesion is localized and the patient’s liver function permits it, but in many cases, complete resection is not possible at the time of diagnosis [66]. For cases where surgery is not feasible or to prevent postoperative recurrence, albendazole is currently the only recommended first-line drug, which inhibits parasite growth by blocking glucose uptake and interfering with mitochondrial function [67]. However, as mentioned earlier, albendazole only has a parasitostatic effect, making it difficult to completely eradicate the parasite, and it requires long-term or even lifelong administration [68]. The effective concentration of the drug in the lesion is extremely low (only 1% of the blood concentration), far below the level required to kill the parasite [69]. Long-term medication also brings adverse reactions such as liver damage and leukopenia, as well as potential drug resistance issues [70]. Therefore, monotherapy often fails to halt disease progression, and recurrence is highly likely upon discontinuation [9]. In response to these challenges, scholars in recent years have actively explored new drugs and strategies, including drug repurposing, targeted immunotherapy, and novel drug delivery systems, with the hope of improving efficacy and reducing toxicity [71]. Below, in conjunction with the characteristics of the AE immune microenvironment, we will discuss the research progress and application prospects of various innovative therapies.

### 6.2. Drug Repurposing and Novel Antiparasitic Drugs (Experimental)

Due to the high cost of developing new drugs and the relatively few AE cases, drug repurposing has become a popular strategy. High-throughput screening has identified multiple marketed drugs that exhibit inhibitory effects on *Echinococcus* in experimental models. For example, the antimalarial drug mefloquine can significantly inhibit parasite growth in infected mice [72]; the proteasome inhibitor bortezomib directly induces protoscolex death in vitro, and in mouse experiments, it reduces the average parasite weight by about 2 g [73]. Additionally, nitroimidazole-containing antibacterials, certain TKIs (tyrosine kinase inhibitors), and others have also shown certain activity [9]. Most of these repurposed drugs already have human safety data, which can accelerate their entry into clinical trials. However, attention must be paid to the evaluation of in vivo efficacy and the dosage safety window. Currently, no new drug has replaced albendazole through clinical validation, but the aforementioned studies provide valuable clues for combination therapies or new treatments.

### 6.3. Emerging Strategies in Immunotherapy (Experimental)

Since AE presents an immune-tolerant microenvironment similar to tumors, some strategies from tumor immunotherapy have been attempted in AE, particularly immune checkpoint inhibitors. Blockade of the PD-1/PD-L1 pathway represents one of the most extensively studied preclinical approaches [74]. In a murine model of chronic AE, anti-PD-L1 monoclonal antibody administration markedly reduced parasite burden and restored effective immune responses, evidenced by increased effector CD4^+^ and CD8^+^ T cells, decreased Treg proportions, and partial recovery of macrophage and dendritic cell function [56]. Notably, PD-L1 monotherapy also attenuated excessive NKT- and NK-cell activation, potentially mitigating immunotherapy-related inflammation. These findings indicate that the PD-1 pathway exerts a critical immunoregulatory role in AE and that its blockade can disrupt parasite-induced immunosuppression, thereby enhancing host control. Analogous preclinical data suggest therapeutic potential for blocking CTLA-4 and TIGIT [57]. Prospective reviews even propose considering antibody therapies targeting immunosuppressive pathways such as PD-1/CTLA-4/TIGIT for inclusion in AE treatment, with the aim of achieving functional clearance of the parasite, rather than mere growth inhibition [75]; however, these immunotherapies have currently only been validated in animal models or in vitro, we emphasize that checkpoint inhibitors are still at an experimental stage in AE—their clinical efficacy and safety remain unproven. Therefore, careful future evaluation of their safety in human AE patients is still needed, particularly to avoid tissue damage caused by overly strong immune responses. In addition to checkpoint blockade, other immunomodulators also deserve attention. For example, inhibiting the IL-33/ST2 axis has shown promise in mice (reducing immunosuppression and fibrosis), but its clinical applicability in AE is untested [12]. Another example is blockers or antibodies targeting the Tim-4 receptor on macrophage surfaces, which are expected to lift its inhibition of pro-inflammatory responses, thereby restoring the body’s anti-parasitic immunity and reducing liver fibrosis. Tim-4 is highly expressed in M2-type macrophages of AE patients, and in vitro downregulation of Tim-4 can increase the release of inflammatory cytokines and reverse some fibrosis indicators, and is thus considered an emerging target for immunotherapy [17]. Furthermore, NKG2A inhibitors targeting NK cells (such as the monoclonal antibody Monalizumab) can theoretically be used to release the “brake” on NK cells, enhancing their direct cytotoxic effects on echinococcal cysts [23,61]. In summary, treating AE as an immune imbalance disease and “rebooting” host immunity through various immunotherapies is an important direction for future treatment.

Caution: Immune-based therapies for AE carry potential risks. Reinvigorating immune responses in an already diseased liver could provoke collateral damage—for example, severe hepatic inflammation or even accelerated lesion progression. Clinical observations show that disruption of immune balance (e.g., prolonged immunosuppression) can dramatically worsen AE [76], highlighting the delicate host–parasite equilibrium. Thus, any checkpoint inhibitor or similar immunotherapy must be pursued judiciously, with careful monitoring to avoid immune-related liver injury.

### 6.4. Novel Drug Delivery Systems (Experimental)

To improve the effective concentration and specificity of drugs at the lesion site, nanocarriers and targeted drug delivery systems also show promise in AE [69]. For instance, researchers have developed nanoparticles to deliver albendazole or other drugs, making them more likely to accumulate in liver lesions and sustain drug release, thereby overcoming the issue of insufficient concentration with traditional oral albendazole [9]. Animal experiments have shown that liposome-encapsulated albendazole significantly increases intrahepatic concentration, with superior efficacy and lower toxicity compared to conventional formulations [77]. In the future, immunotherapy drugs (such as checkpoint inhibitors) and antiparasitic drugs can be co-loaded into bifunctional nanocarriers to achieve synchronous intervention on the parasite and its immune microenvironment [78].

### 6.5. Traditional/Natural Approaches and Vaccines (Experimental)

The roles of traditional Chinese medicine and natural products in immune regulation and antiparasitic effects have attracted attention [79]. For example, flavonoids, terpenoids, and other compounds have shown certain anti-echinococcal activity and can influence macrophage function [80]. Finally, in terms of vaccine prevention, there is currently no AE vaccine for human use, but experimental vaccines (such as recombinant *Echinococcus* antigen Eg95) in intermediate hosts (rodents) have shown certain protective efficacy [81]. If an effective human AE vaccine can be developed and administered to populations in endemic areas, it will reduce new cases at the source. However, due to the complex life cycle and immune evasion strategies of *Echinococcus*, vaccine development still faces many challenges [82,83].

Taken together, current AE management still relies mainly on surgery and benzimidazole therapy, both of which have important limitations. Emerging approaches, including immunomodulation, drug repurposing, targeted delivery systems, and vaccines, remain largely preclinical but may offer future therapeutic opportunities. The existing and potential therapeutic strategies for the aforementioned AE are summarized in Table 3.

## 7. Conclusions

Alveolar echinococcosis is highly aggressive; its key pathogenesis lies in constructing a unique immunosuppressive microenvironment, allowing the parasite to colonize and expand long-term in the host’s liver while the host still finds it difficult to completely eliminate it [84]. This article reviews the research progress on hepatic macrophage polarization and immune microenvironment remodeling in AE infection, showing that the functional shift in macrophages from M1 to M2 permeates the disease’s occurrence and development process [38]. At the same time, it should be acknowledged that the M1/M2 classification represents a simplified conceptual framework and does not fully capture the phenotypic diversity of macrophages in vivo. Accordingly, therapeutic strategies aimed at shifting macrophages from an M2-like to an M1-like state should be interpreted with caution. Given the presence of intermediate and context-dependent phenotypes, more effective approaches may require targeting specific macrophage functions, such as profibrotic activity or immunoregulatory cytokine production, rather than assuming a uniform binary transition. The parasite promotes macrophage polarization to an immune-tolerant phenotype by secreting antigenic molecules (such as phytate, exosomal miRNAs, etc.) and inducing the release of host factors (such as IL-33), and further recruits Treg, exhausts effector T cells, and other inhibitory components through intercellular signaling networks, gradually establishing an “immune sanctuary” similar to a tumor microenvironment [12,41,44,85,86]. This microenvironment on one hand protects the parasite from immune clearance, and on the other hand causes chronic inflammation and fibrotic damage to host tissues [87,88]. Recent extensive studies have revealed key molecules and cellular interactions in this process: from the RhoA-MAPK signaling pathway in macrophages to PD-1/CTLA-4 checkpoints in T cells, from Tim-4-mediated apoptotic cell clearance to IL-33-driven Th2 deviation, each link may become a target for intervention [12,16,17,22]. Looking to the future, AE treatment is expected to transcend the limitations of existing antiparasitic drugs, shifting towards a comprehensive strategy that addresses both “parasite + microenvironment.” In particular, the introduction of immunotherapy offers a promising new avenue for refractory AE [89]. If surgical resection, targeted antiparasitic drugs, and immune microenvironment modulators can be organically combined, it may achieve functional cure for AE, improving patient survival rates and quality of life [90]. In summary, in-depth research on the immunological mechanisms of AE not only enriches our understanding of immune evasion phenomena in parasitic infections but also lays a theoretical foundation for developing more effective intervention measures. While much of the mechanistic understanding of AE macrophage polarization is derived from animal models and in vitro experiments, with relatively few confirmations in human tissue—thus, our conclusions should be viewed in light of these limitations. So future research should further focus on the regulation of key immune nodes and their clinical translation evaluations, promoting the field from basic research to clinical applications, contributing to conquering the intractable disease of alveolar echinococcosis.

## Figures and Tables

**Table 1:** Summary of Molecular Mechanisms by Which *E. multilocularis* Induces M2 Polarization.

Inducing Factor	Mechanism	Effect	Reference	Evidence Type
Phytic acid (from laminated layer)	Suppresses IL-6/IL-1β production via macrophage signaling inhibition.	Promotes M2 shift, reduces inflammation, aids parasite persistence.	[41]	in vitro macrophage experiments/mouse models
Exosomal miRNAs (e.g., predicted miR-10a-5p from *E. multilocularis* EVs)	Targets STAT6/PPARγ or LIF/JAK1-STAT3 pathways to inhibit M1 and promote M2.	Alleviates inflammation; potential AE immune evasion.	[43,44]	non-AE-related evidence
Parasite antigens (e.g., Antigen B from analogous *E. granulosus*)	Regulates TLR4 endocytosis, inhibits phagocytosis.	Drives M2 polarization, immunosuppression, fibrosis.	[42,45]	non-AE-related evidence
Soluble antigens/Cyst fluid components	Activates RhoA/MAPK pathways; induces IL-10/TGF-β secretion.	Enhances M2 phenotype, T-cell exhaustion, angiogenesis/fibrosis.	[16,40]	in vitro macrophage experiments
Protoscolex components (e.g., from vesicle fluid)	Direct interaction suppressing NK/DC functions; promotes Treg expansion.	Reshapes immune microenvironment toward tolerance.	[46,47]	in vitro macrophage experiments
Glycolytic reprogramming factors (parasite-derived metabolites)	Activates PI3K/Akt/mTOR pathways; shifts metabolism to support M2 survival.	Promotes angiogenesis and fibrosis in AE lesions.	[39,48]	in vitro macrophage experiments

**Table 2:** Remodeling of the Immune Microenvironment in AE.

Component	Role in Remodeling	Key Interactions	Reference	Evidence Type
M2 Macrophages	Recruit Tregs, suppress NK/DC functions via IL-10/TGF-β.	Foster angiogenesis/fibrosis; promote T-cell exhaustion.	[40,53]	mouse models/human tissue observations
Regulatory T Cells (Treg)	Downregulate Th1/Th17 via IL-10/TGF-β; expand under parasite influence.	Establish immune tolerance; correlate with chronic infection.	[58,59]	mouse models/human tissue observations
Immune checkpoints (e.g., PD-1/PD-L1)	Induce T-cell exhaustion; upregulated in AE lesions.	Suppress effector immunity; potential therapeutic target.	[56,57]	mouse models/human tissue observations
Chemokines (e.g., CCL17/CCL19)	Markers of disease progression; recruit immunosuppressive cells.	Modulate macrophage infiltration and fibrosis.	[51,52]	human tissue observations/mouse models
NK cells and exhaustion markers (e.g., NKG2A, Tim-3)	Impaired function in lesions; high exhaustion expression.	Contribute to parasite persistence; reversible in models.	[60,61]	mouse models/human tissue observations
Fibrotic factors (e.g., Wnt signaling)	Activate epithelial–mesenchymal transition; promote hepatic fibrosis.	Interact with M2 macrophages; exacerbate tumor-like niche.	[39,52]	mouse models/human tissue observations

**Table 3:** Existing and Potential Treatment Strategies for AE.

Strategy	Description	Potential Benefits/Risks	Reference	Evidence Type
Surgical Resection	Radical removal when feasible; combined with drugs.	High cure rate in early stages; recurrence risk if incomplete.	[66,67]	clinical validation
Benzimidazole therapy (e.g., albendazole)	Parasitostatic effects; long-term administration for inoperable cases.	Controls growth but not curative; hepatotoxicity risk.	[65,70]	clinical validation
Drug repurposing (e.g., mefloquine, bortezomib)	Anti-malarial/proteasome inhibitors against metacestodes.	Enhanced efficacy in models; limited clinical data.	[72,73]	experimental concept
Immunotherapy (e.g., PD-1/CTLA-4/TIGIT blockade)	Restore effector immunity; reverse exhaustion in models.	Promising preclinical; risks of inflammation exacerbation.	[74,84]	experimental concept (preclinical only)
Nanodelivery systems (e.g., PLGA nanoparticles)	Targeted albendazole delivery to liver.	Improved bioavailability; reduced side effects.	[69,78]	experimental concept
Vaccines (e.g., multi-epitope like EG95 or GILE)	Induce protective immunity against infection.	Preventive potential; efficacy in animal models.	[81,83]	experimental concept
Traditional Chinese Medicine and Natural Products (e.g., Flavonoids, terpenoids)	anti-echinococcal activity and can affect macrophage function.	Holds promise as adjunctive treatment methods, requiring further clarification of effective components and mechanisms of action.	[79,80]	experimental concept

## Data Availability

No new data were created or analyzed in this study.

## Abbreviations

The following abbreviations are used in this manuscript:

AE	Alveolar echinococcosis
Th1	T cell helper 1
Th17	T cell helper 17
Th2	T cell helper 2
Treg	Regulatory T cells
IL-1β	Interleukin-1 beta
IL-12	Interleukin-12
TNF-α	Tumor necrosis factor-alpha
IFN-γ	Interferon-gamma
NK	Natural killer
IL-17	Interleukin-17
IgG	Immunoglobulin G
IgE	Immunoglobulin E
M1	Classically activated macrophages
M2	Alternatively activated macrophages
LPS	Lipopolysaccharide
STAT1	Signal transducer and activator of transcription 1
NF-κB	Nuclear factor kappa B
iNOS	Inducible nitric oxide synthase
Arg-1	Arginase-1
CD206	Cluster of differentiation 206
PPARγ	Peroxisome proliferator-activated receptor gamma
GM-CSF	Granulocyte-macrophage colony-stimulating factor
IRF5	Interferon regulatory factor 5
IRF8	Interferon regulatory factor 8
IL-4	Interleukin-4
IL-13	Interleukin-13
M-CSF	Macrophage colony-stimulating factor
PI3K	Phosphoinositide 3-kinase
Akt	Protein kinase B
KLF4	Kruppel-like factor 4
JMJD3	Jumonji domain-containing protein 3
Fizz1	Found in inflammatory zone 1
Ym1	Chitinase-like protein
IL-10	Interleukin-10
TGF-β	Transforming growth factor-beta
HIF-1α	Hypoxia-inducible factor 1-alpha
HSC	Hepatic stellate cells
α-SMA	Alpha-smooth muscle actin
CCL22	C-C motif chemokine ligand 22
HK	Hexokinase
PFK	Phosphofructokinase
EVs	Extracellular vesicles
AgB	Antigen B
Fcγ	Fc gamma
let-7-5p	MicroRNA let-7-5p
miR-10a-5p	MicroRNA-10a-5p
IL-33	Interleukin-33
ST2	Suppression of Tumorigenicity 2
FOXP3	Forkhead box P3
CCL17	C-C motif chemokine ligand 17
CCL19	C-C motif chemokine ligand 19
CLT	Close liver tissue
DLT	Distant liver tissue
DC	Dendritic cells
PDGF	Platelet-derived growth factor
PD-1	Programmed death-1
CTLA-4	Cytotoxic T-lymphocyte-associated protein 4
TIGIT	T cell immunoreceptor with Ig and ITIM domains
Tim-3	T cell immunoglobulin and mucin domain-3
CD4	Cluster of differentiation 4
CD8	Cluster of differentiation 8
CD25	Cluster of differentiation 25
NKG2A	Natural killer group 2A
HLA-E	Human leukocyte antigen-E
TLR	Toll-like receptor
VEGF	Vascular endothelial growth factor
Tfh	T follicular helper
Tim-4	T cell immunoglobulin and mucin domain-4
NOTCH	Notch signaling pathway
LAG-3	Lymphocyte activation gene-3
OXPHOS	Oxidative phosphorylation
BMP9	Bone morphogenetic protein 9
ID1	Inhibitor of DNA binding 1
VEGFA	Vascular endothelial growth factor A
WHO	World Health Organization

## References

[1] Deplazes P., Rinaldi L., Alvarez Rojas C.A., Torgerson P.R., Harandi M.F., Romig T., Antolova D., Schurer J.M., Lahmar S., Cringoli G. (2017). Global Distribution of Alveolar and Cystic Echinococcosis. Adv. Parasitol..

[2] Wen H., Vuitton L., Tuxun T., Li J., Vuitton D.A., Zhang W., McManus D.P. (2019). Echinococcosis: Advances in the 21st Century. Clin. Microbiol. Rev..

[3] Aji T., Dong J.H., Shao Y.M., Zhao J.M., Li T., Tuxun T., Shalayiadang P., Ran B., Jiang T.M., Zhang R.Q. (2018). Ex vivo liver resection and autotransplantation as alternative to allotransplantation for end-stage hepatic alveolar echinococcosis. J. Hepatol..

[4] Ferrer Inaebnit E., Molina Romero F.X., Segura Sampedro J.J., González Argenté X., Morón Canis J.M. (2022). A review of the diagnosis and management of liver hydatid cyst. Rev. Española Enfermedades Dig..

[5] Ma T., Wang Q., Hao M., Xue C., Wang X., Han S., Wang Q., Zhao J., Ma X., Wu X. (2023). Epidemiological characteristics and risk factors for cystic and alveolar echinococcosis in China: An analysis of a national population-based field survey. Parasites Vectors.

[6] Tamarozzi F., Ronzoni N., Degani M., Oliboni E., Tappe D., Gruener B., Gobbi F. (2024). Confirmed Autochthonous Case of Human Alveolar Echinococcosis, Italy, 2023. Emerg. Infect. Dis..

[7] Shafiei R., Mohajerzadeh M.S., Masomi H.F.A., Tavakoli M., Turki H., Firouzeh N. (2024). Discordance Therapeutic Protocol of Cystic Echinococcosis with WHO Guideline: A Descriptive Study Based on Liver Ultra-Sonographic Data in North Khorasan Province, Northeastern of Iran. J. Ultrasound Med..

[8] Meilinger M., Stoeckl C., Pollheimer M., Kern P., Reisinger E.C., Seeber K., Krause R., Flick H., Hoenigl M. (2013). Progressive alveolar echinococcosis after discontinuation of anthelmintic therapy. Parasites Vectors.

[9] Xu X., Qian X., Gao C., Pang Y., Zhou H., Zhu L., Wang Z., Pang M., Wu D., Yu W. (2022). Advances in the pharmacological treatment of hepatic alveolar echinococcosis: From laboratory to clinic. Front. Microbiol..

[10] Qian M.B., Abela-Ridder B., Wu W.P., Zhou X.N. (2017). Combating echinococcosis in China: Strengthening the research and development. Infect. Dis. Poverty.

[11] Casulli A. (2020). Recognising the substantial burden of neglected pandemics cystic and alveolar echinococcosis. Lancet Glob. Health.

[12] Autier B., Manuel C., Lundstroem-Stadelmann B., Girard J.P., Gottstein B., Gangneux J.P., Samson M., Robert-Gangneux F., Dion S. (2023). Endogenous IL-33 Accelerates Metacestode Growth during Late-Stage Alveolar Echinococcosis. Microbiol. Spectr..

[13] Gao H., Huo L., Mo X., Jiang B., Luo Y., Xu B., Li J., Ma X., Jing T., Feng Z. (2022). Suppressive effect of pseudolaric acid B on Echinococcus multilocularis involving regulation of TGF-β1 signaling in vitro and in vivo. Front. Microbiol..

[14] Zhang C., Lin R., Li Z., Yang S., Bi X., Wang H., Aini A., Zhang N., Abulizi A., Sun C. (2020). Immune Exhaustion of T Cells in Alveolar Echinococcosis Patients and Its Reversal by Blocking Checkpoint Receptor TIGIT in a Murine Model. Hepatology.

[15] Wang J., Gottstein B. (2016). Immunoregulation in larval Echinococcus multilocularis infection. Parasite Immunol..

[16] Chong S., Chen G., Dang Z., Niu F., Zhang L., Ma H., Zhao Y. (2022). Echinococcus multilocularis drives the polarization of macrophages by regulating the RhoA-MAPK signaling pathway and thus affects liver fibrosis. Bioengineered.

[17] Wang L., Liu Y., Ma Y., Zhou X., Aibibula M., Zhang X., Zhao H., Zhou J., Tian F., Ma X. (2025). TIM4+macrophages suppress the proinflammatory response to maintain the chronic alveolar echinococcosis infection. Front. Cell. Infect. Microbiol..

[18] Vuitton D.A., Zhang S.L., Yang Y., Godot V., Beurton I., Mantion G., Bresson-Hadni S. (2006). Survival strategy of Echinococcus multilocularis in the human host. Parasitol. Int..

[19] Nian X., Li L., Ma X., Li X., Li W., Zhang N., Ohiolei J.A., Li L., Dai G., Liu Y. (2022). Understanding pathogen-host interplay by expression profiles of lncRNA and mRNA in the liver of Echinococcus multilocularis-infected mice. PLoS Neglected Trop. Dis..

[20] Gottstein B., Wang J., Boubaker G., Marinova I., Spiliotis M., Müller N., Hemphill A. (2015). Susceptibility versus resistance in alveolar echinococcosis (larval infection with Echinococcus multilocularis). Vet. Parasitol..

[21] Wang J., Müller S., Lin R., Siffert M., Vuitton D.A., Wen H., Gottstein B. (2017). Depletion of FoxP3(+) Tregs improves control of larval Echinococcus multilocularis infection by promoting co-stimulation and Th1/17 immunity. Immun. Inflamm. Dis..

[22] Sun T., Yang Y., Qiu Y., Wang T., Yang M., Shen S., Wang W. (2024). High PD-1 and CTLA-4 expression correlates with host immune suppression in patients and a mouse model infected with Echinococcus multilocularis. Parasites Vectors.

[23] Abulizi A., Tuergan T., Shalayiadang P., Zhang C., Zhang R., Jiang T., Guo Q., Wang H., Li L., Lin R. (2025). Correction: Hepatic alveolar echinococcosis infection induces a decrease in NK cell function through high expression of NKG2A in patients. Front. Immunol..

[24] Feng C., Cheng Z., Xu Z., Tian Y., Tian H., Liu F., Luo D., Wang Y. (2022). EmCyclinD-EmCDK4/6 complex is involved in the host EGF-mediated proliferation of Echinococcus multilocularis germinative cells via the EGFR-ERK pathway. Front. Microbiol..

[25] Colin S., Chinetti-Gbaguidi G., Staels B. (2014). Macrophage phenotypes in atherosclerosis. Immunol. Rev..

[26] Martinez F.O., Helming L., Gordon S. (2009). Alternative activation of macrophages: An immunologic functional perspective. Annu. Rev. Immunol..

[27] Abdelaziz M.H., Abdelwahab S.F., Wan J., Cai W., Huixuan W., Jianjun C., Kumar K.D., Vasudevan A., Sadek A., Su Z. (2020). Alternatively activated macrophages; a double-edged sword in allergic asthma. J. Transl. Med..

[28] Strizova Z., Benesova I., Bartolini R., Novysedlak R., Cecrdlova E., Foley L.K., Striz I. (2023). M1/M2 macrophages and their overlaps–myth or reality?. Clin. Sci..

[29] Cerdeira C.D., Brigagão M. (2024). Targeting Macrophage Polarization in Infectious Diseases: M1/M2 Functional Profiles, Immune Signaling and Microbial Virulence Factors. Immunol. Investig..

[30] Zhang T., Zhang Y., Yang Z., Jiang Y., Sun L., Huang D., Tian M., Shen Y., Deng J., Hou J. (2023). Echinococcus multilocularis protoscoleces enhance glycolysis to promote M2 Macrophages through PI3K/Akt/mTOR Signaling Pathway. Pathog. Glob. Health.

[31] Raes G., De Baetselier P., Noël W., Beschin A., Brombacher F., Hassanzadeh Gh G. (2002). Differential expression of FIZZ1 and Ym1 in alternatively versus classically activated macrophages. J. Leukoc. Biol..

[32] Wang S., Liu G., Li Y., Pan Y. (2022). Metabolic Reprogramming Induces Macrophage Polarization in the Tumor Microenvironment. Front. Immunol..

[33] Shen Y., Zhang T., Yang Z., Zhang Y., Huang D., Hou J., Tian M., Ma Y. (2024). Preliminary study on the effect of Echinococcus multilocaris on phenotypic transformations of glucose metabolism and polarization types in macrophages. Zhongguo Xue Xi Chong Bing. Fang. Zhi Za Zhi.

[34] Labonte A.C., Sung S.J., Jennelle L.T., Dandekar A.P., Hahn Y.S. (2017). Expression of scavenger receptor-AI promotes alternative activation of murine macrophages to limit hepatic inflammation and fibrosis. Hepatology.

[35] Ma P., Zhao W., Gao C., Liang Z., Zhao L., Qin H., Sun X. (2022). The Contribution of Hepatic Macrophage Heterogeneity during Liver Regeneration after Partial Hepatectomy in Mice. J. Immunol. Res..

[36] Wang C., Ma C., Gong L., Guo Y., Fu K., Zhang Y., Zhou H., Li Y. (2021). Macrophage Polarization and Its Role in Liver Disease. Front. Immunol..

[37] Moore M.P., Cunningham R.P., Davis R.A.H., Deemer S.E., Roberts B.M., Plaisance E.P., Rector R.S. (2021). A dietary ketone ester mitigates histological outcomes of NAFLD and markers of fibrosis in high-fat diet fed mice. Am. J. Physiol.-Gastrointest. Liver Physiol..

[38] Ma Y., Li J., Liu Y., Zhao H., Qi X., Sun Y., Chen J., Zhou J., Ma X., Wang L. (2025). Corrigendum to “Identification and exploration of a new M2 macrophage marker MTLN in alveolar echinococcosis” [International Immunopharmacology 131 (2024) 111808]. Int. Immunopharmacol..

[39] Wu B., Yang Y., Cheng S., Wang Z., Fan H. (2025). Activation of the Wnt signaling pathway and its role in epithelial-mesenchymal transition and hepatic fibrosis in alveolar echinococcosis. Front. Cell. Infect. Microbiol..

[40] Wang H., Zhang C.S., Fang B.B., Hou J., Li W.D., Li Z.D., Li L., Bi X.J., Li L., Abulizi A. (2020). Dual Role of Hepatic Macrophages in the Establishment of the Echinococcus multilocularis Metacestode in Mice. Front. Immunol..

[41] Salzmann M., Resch U., Boccuni L., Schneider C., Pichler E.T., Brekalo M., Uhrin P., Kronenberg P.A., Wassermann M., Romig T. (2025). Phytic acid impairs macrophage inflammatory response in Echinococcus multilocularis infection. Commun. Biol..

[42] Zhang Y., Yue Y., Cheng Y., Jiao H., Yan M. (2025). Antigen B from Echinococcus granulosus regulates macrophage phagocytosis by controlling TLR4 endocytosis in immune thrombocytopenia. Chem. Biol. Interact..

[43] Liu Y., Cai Y.C., Chen J.X., Chen S.H., Yu Y.F. (2025). miRNA let-7-5p present in the extracellular vesicles of Trichinella spiralis newborn larvae inhibits the function of M1-type RAW264.7 macrophages by targeting C/EBPδ. Parasites Vectors.

[44] Xin Y., Wen R., Song D., Xiao J., Gao X., Yin M., Bai Y., Wang J., Zhou X., Zhao J. (2025). Emu-miR-10a-5p in Echinococcus multilocularis-derived-extracellular vesicles alleviates airway inflammation in mice with allergic asthma by inhibiting macrophage M2a polarization through LIF-mediated JAK1-STAT3 signaling. Front. Immunol..

[45] Virginio V.G., Taroco L., Ramos A.L., Ferreira A.M., Zaha A., Ferreira H.B., Hernández A. (2007). Effects of protoscoleces and AgB from Echinococcus granulosus on human neutrophils: Possible implications on the parasite’s immune evasion mechanisms. Parasitol. Res..

[46] Reinehr M., Micheloud C., Grimm F., Kronenberg P.A., Grimm J., Beck A., Nell J., Meyer Zu Schwabedissen C., Furrer E., Müllhaupt B. (2020). Pathology of Echinococcosis: A Morphologic and Immunohistochemical Study on 138 Specimens with Focus on the Differential Diagnosis Between Cystic and Alveolar Echinococcosis. Am. J. Surg. Pathol..

[47] Bellanger A.P., Courquet S., Pallandre J.R., Godet Y., Millon L. (2020). Echinococcus multilocularis vesicular fluid induces the expression of immune checkpoint proteins in vitro. Parasite Immunol..

[48] Ke F., Xu M.Z., Ma L., Chen Q.D., He B.B., A J.D. (2024). Progress and perspectives on BMP9-ID1 activation of HIF-1α and VEGFA to promote angiogenesis in hepatic alveolar echinococcosis. Front. Oncol..

[49] Yang Q., Jia W., Wang X., Cai Q., Ge X., Wang W., Han X. (2023). Single-cell RNA sequencing deciphers transcriptional profiles of hepatocytes in mouse with hepatic alveolar echinococcosis. Zhongguo Xue Xi Chong Bing. Fang. Zhi Za Zhi.

[50] Wang H., Li Y., Yu Q., Wang M., Ainiwaer A., Tang N., Zheng X., Duolikun A., Deng B., Li J. (2024). Immunological Characteristics of Hepatic Dendritic Cells in Patients and Mouse Model with Liver Echinococcus multilocularis Infection. Trop. Med. Infect. Dis..

[51] Chen J., Ma Y., Liu Y., Zhao H., Qi X., Sun Y., Zhou X., Zhou J., Ma X., Wang L. (2024). CCL17 and CCL19 are markers of disease progression in alveolar echinococcosis. Cytokine.

[52] Wang Z., Du K., Jin N., Tang B., Zhang W. (2023). Macrophage in liver Fibrosis: Identities and mechanisms. Int. Immunopharmacol..

[53] Liu Y., Tian F., Shan J., Gao J., Li B., Lv J., Zhou X., Cai X., Wen H., Ma X. (2020). Kupffer Cells: Important Participant of Hepatic Alveolar Echinococcosis. Front. Cell. Infect. Microbiol..

[54] Salnikov M., Prusinkiewicz M.A., Lin S., Ghasemi F., Cecchini M.J., Mymryk J.S. (2023). Tumor-Infiltrating T Cells in EBV-Associated Gastric Carcinomas Exhibit High Levels of Multiple Markers of Activation, Effector Gene Expression, and Exhaustion. Viruses.

[55] Zhang X., Li L., Sun T., Yang N., Liu H., Chu J., Xue J., Lü G., Aji T., Bi X. (2025). CD155 as a therapeutic target in alveolar echinococcosis: Insights from an Echinococcus multilocularis infection mouse model. Front. Microbiol..

[56] Jebbawi F., Bellanger A.P., Lunström-Stadelmann B., Rufener R., Dosch M., Goepfert C., Gottstein B., Millon L., Grandgirard D., Leib S.L. (2021). Innate and adaptive immune responses following PD-L1 blockade in treating chronic murine alveolar echinococcosis. Parasite Immunol..

[57] Yang Y., Wuren T., Wu B., Cheng S., Fan H. (2024). The expression of CTLA-4 in hepatic alveolar echinococcosis patients and blocking CTLA-4 to reverse T cell exhaustion in Echinococcus multilocularis-infected mice. Front. Immunol..

[58] Li D., Ainiwaer A., Zheng X., Wang M., Shi Y., Rousu Z., Hou X., Kang X., Maimaiti M., Wang H. (2023). Upregulation of LAG3 modulates the immune imbalance of CD4+ T-cell subsets and exacerbates disease progression in patients with alveolar echinococcosis and a mouse model. PLoS Pathog..

[59] Nono J.K., Lutz M.B., Brehm K. (2020). Expansion of Host Regulatory T Cells by Secreted Products of the Tapeworm Echinococcus multilocularis. Front. Immunol..

[60] Zhao H., Li B., Pang N., Li Z., Aibibula M., Tian F., Cai X., Li Y., Ding J., Ma X. (2021). High Expression of Tim-3 in Alveolar Echinococcosis Mediates Depletion of CD8(+) T Cell Function. Ann. Clin. Lab. Sci..

[61] Aierken A., Aierken A., Aimulajiang K., Abulizi A., Yuan Z., Liu C., Zhu D., Zhao H., Aji T. (2025). Blocking NKG2A in Echinococcus multilocularis infection partially relieves impairment of NK cell function of the host. Cell. Mol. Life Sci..

[62] Zhang H., Meng R., Zhang F., Chen A., Ge H., Chen W., Li Z., Fu Y. (2025). IDO1 promotes Echinococcus multilocularis infection by regulating the formation of neutrophil extracellular traps. Vet. Res..

[63] Bakhtiar N.M., Spotin A., Mahami-Oskouei M., Ahmadpour E., Rostami A. (2020). Recent advances on innate immune pathways related to host-parasite cross-talk in cystic and alveolar echinococcosis. Parasites Vectors.

[64] Manabe M., Nakamura T., Sato K., Hayashi N., Kouguchi H., Nakao R., Hidaka M., Matsuyama H., Nonaka N., Morimatsu M. (2025). Efferocytosis-Driven M2 Macrophage Impairs Fibrotic Encapsulation and Promotes Echinococcus multilocularis Growth in Cotton Rats (Sigmodon hispidus). Microsc. Microanal..

[65] Calame P., Richou C., Blagosklonov O., Grenouillet F., Heurgué A., Villena I., Frentiu E., Turco C., Simon G., Vuitton D.A. (2025). Is a shortened postoperative albendazole duration after curative surgery for alveolar echinococcosis possible? Results from a prospective multicenter study. Parasites Vectors.

[66] Plum P.E., Ausselet N., Kidd F., Noirhomme S., Garrino M.G., Dili A., Hayette M.P., Detry O., Leonard P., Motet C. (2025). EchiNam: Multicenter retrospective study on the experience, challenges, and pitfalls in the diagnosis and treatment of alveolar echinococcosis in Belgium. Eur. J. Clin. Microbiol. Infect. Dis..

[67] Jing Q.D., A J.D., Liu L.X., Fan H.N. (2024). Current status of drug therapy for alveolar echinococcosis. World J. Hepatol..

[68] Zhou Z., Huayu M., Mu Y., Tang F., Ge R.L. (2024). Ubenimex combined with Albendazole for the treatment of Echinococcus multilocularis-induced alveolar echinococcosis in mice. Front. Vet. Sci..

[69] Li J., Yang Y., Han X., Li J., Tian M., Qi W., An H., Wu C., Zhang Y., Han S. (2023). Oral Delivery of Anti-Parasitic Agent-Loaded PLGA Nanoparticles: Enhanced Liver Targeting and Improved Therapeutic Effect on Hepatic Alveolar Echinococcosis. Int. J. Nanomed..

[70] Deibel A., Stocker D., Meyer Zu Schwabedissen C., Husmann L., Kronenberg P.A., Grimm F., Deplazes P., Reiner C.S., Müllhaupt B. (2022). Evaluation of a structured treatment discontinuation in patients with inoperable alveolar echinococcosis on long-term benzimidazole therapy: A retrospective cohort study. PLoS Neglected Trop. Dis..

[71] Wang W., Li J., Qi W., Chen Y., Tian M., Wu C., Zhang Y., Yu Y., Han S., Han X. (2024). Drug repurposing for hard-to-treat human alveolar echinococcosis: Pyronaridine and beyond. Parasitology.

[72] Lundström-Stadelmann B., Rufener R., Hemphill A. (2020). Drug repurposing applied: Activity of the anti-malarial mefloquine against Echinococcus multilocularis. Int. J. Parasitol. Drugs Drug Resist..

[73] Stadelmann B., Aeschbacher D., Huber C., Spiliotis M., Müller J., Hemphill A. (2014). Profound activity of the anti-cancer drug bortezomib against Echinococcus multilocularis metacestodes identifies the proteasome as a novel drug target for cestodes. PLoS Neglected Trop. Dis..

[74] Zhang C., Wang H., Aji T., Li Z., Li Y., Ainiwaer A., Rousu Z., Li J., Wang M., Deng B. (2024). Targeting myeloid-derived suppressor cells promotes antiparasitic T-cell immunity and enhances the efficacy of PD-1 blockade (15 words). Nat. Commun..

[75] Autier B., Robert-Gangneux F., Dion S. (2024). Chemotherapy for the treatment of alveolar echinococcosis: Where are we?. Parasite.

[76] Diem S., Gottstein B., Beldi G., Semmo N., Diem L.F. (2021). Accelerated Course of Alveolar Echinococcosis After Treatment with Steroids in a Patient with Autoimmune Encephalitis. Cureus.

[77] Wen H., New R.R., Muhmut M., Wang J.H., Wang Y.H., Zhang J.H., Shao Y.M., Craig P.S. (1996). Pharmacology and efficacy of liposome-entrapped albendazole in experimental secondary alveolar echinococcosis and effect of co-administration with cimetidine. Parasitology.

[78] Gong Y., Zhou T., Ma R., Yang J., Zhao Y., Pan M., Huang Z., Wen H., Jiang H., Wang J. (2024). Efficacy and mechanism of energy metabolism dual-regulated nanoparticles (atovaquone-albendazole nanoparticles) against cystic echinococcosis. BMC Infect. Dis..

[79] Newman D.J., Cragg G.M. (2020). Natural Products as Sources of New Drugs over the Nearly Four Decades from 01/1981 to 09/2019. J. Nat. Prod..

[80] Ali R., Khan S., Khan M., Adnan M., Ali I., Khan T.A., Haleem S., Rooman M., Norin S., Khan S.N. (2020). A systematic review of medicinal plants used against Echinococcus granulosus. PLoS ONE.

[81] Korhonen P.K., Kinkar L., Young N.D., Cai H., Lightowlers M.W., Gauci C., Jabbar A., Chang B.C.H., Wang T., Hofmann A. (2022). Chromosome-scale Echinococcus granulosus (genotype G1) genome reveals the Eg95 gene family and conservation of the EG95-vaccine molecule. Commun. Biol..

[82] Jiang T., Mahemuti M., Wang W., Han S., Wu X., Liu H., Chen Q., Mo X., Wang X., Kadiaili A. (2025). Development and protective efficacy of multi-epitope vaccine FL46 against cystic echinococcosis. Front. Immunol..

[83] Zhou P., Zhou Z., Huayu M., Wang L., Feng L., Xiao Y., Dai Y., Xin M., Tang F., Li R. (2022). A multi-epitope vaccine GILE against Echinococcus Multilocularis infection in mice. Front. Immunol..

[84] Wang J., Jebbawi F., Bellanger A.P., Beldi G., Millon L., Gottstein B. (2018). Immunotherapy of alveolar echinococcosis via PD-1/PD-L1 immune checkpoint blockade in mice. Parasite Immunol..

[85] Zheng Y., Guo X., He W., Shao Z., Zhang X., Yang J., Shen Y., Luo X., Cao J. (2016). Effects of Echinococcus multilocularis miR-71 mimics on murine macrophage RAW264.7 cells. Int. Immunopharmacol..

[86] Guo X., Zheng Y. (2020). Profiling of miRNAs in Mouse Peritoneal Macrophages Responding to Echinococcus multilocularis Infection. Front. Cell. Infect. Microbiol..

[87] Jiang T., Sun W., Aji T., Shao Y., Guo C., Zhang C., Ran B., Hou J., Yasen A., Guo Q. (2022). Single-Cell Heterogeneity of the Liver-Infiltrating Lymphocytes in Individuals with Chronic Echinococcus multilocularis Infection. Infect. Immun..

[88] Li B., Wang L., Qi X., Liu Y., Li J., Lv J., Zhou X., Cai X., Shan J., Ma X. (2023). NOTCH signaling inhibition after DAPT treatment exacerbates alveolar echinococcosis hepatic fibrosis by blocking M1 and enhancing M2 polarization. FASEB J..

[89] Wang S., Ma Y., Wang W., Dai Y., Sun H., Li J., Wang S., Li F. (2022). Status and prospect of novel treatment options toward alveolar and cystic echinococcosis. Acta Trop..

[90] Hataminejad M., Anvari D., Khaleghi N., Nayeri T., Shirazinia R., Shariatzadeh S.A., Hosseini S.A., Siyadatpanah A., Gholami S. (2024). Current status and future prospects of Echinococcus multilocularis vaccine candidates: A systematic review. Vet. Anim. Sci..

